# Clinical outcomes and risk stratification in unresectable biliary tract cancers undergoing radiation therapy

**DOI:** 10.1186/s13014-024-02481-y

**Published:** 2024-08-01

**Authors:** Uri Amit, Misra Shagun, John P. Plastaras, James M. Metz, Thomas B. Karasic, Maryanne J. Lubas, Edgar Ben-Josef

**Affiliations:** 1 Department of Radiation Oncology, Perelman School of Medicine, University of Pennsylvania, Philadelphia, PA USA; 2 Department of Radiation Oncology, Tel Aviv Medical Center, Tel Aviv, Israel; 3 Department of Radiotherapy, Sanjay Gandhi Postgraduate Institute of Medical Sciences, Lucknow, India; 4 Department of Hematology and Oncology, Perelman School of Medicine, University of Pennsylvania, Philadelphia, PA USA; 5 Department of Radiation Oncology, Fox Chase Cancer Center, Philadelphia, PA USA

**Keywords:** Biliary tract cancers, Radiation therapy, CA19-9, Albumin-bilirubin grade, Survival

## Abstract

**Background:**

Biliary tract cancers (BTC) are rare and aggressive malignancies originating from intrahepatic and extrahepatic bile ducts and the gallbladder. Surgery is the only curative option, but due to late-stage diagnosis, is frequently not feasible, leaving chemotherapy as the primary treatment. Radiotherapy (RT) can be an effective alternative for patients with unresectable, non-metastatic BTC despite the generally poor prognosis and significant variability. To help manage patients with unresectable BTC who receive RT, we aimed to identify prognostic markers that could aid in predicting overall survival (OS).

**Methods:**

A retrospective cohort study was conducted at the University of Pennsylvania, involving seventy-eight patients with unresectable BTC treated with definitive intent RT. Comprehensive demographic, clinical, and treatment-related data were extracted from the electronic medical records. Univariate and multivariate Cox regressions were employed to identify predictors of OS after RT. A biomarker model was developed for refined survival prediction.

**Results:**

The cohort primarily comprised patients with good performance status without significant hepatic dysfunction at presentation. The predominant treatment approach involved hypofractionated RT or concurrent 5FU-based chemoRT. Median OS after RT was 12.3 months, and 20 patients (15.6%) experienced local progression with a median time of 30.1 months. Univariate and multivariate analyses identified CA19-9 (above median) and higher albumin-bilirubin (ALBI) grades at presentation as significant predictors of poor OS. Median OS after RT was 24 months for patients with no risk factors and 6.3 months for those with both.

**Conclusions:**

Our study demonstrates generally poor but significantly heterogeneous OS in patients with unresectable BTC treated with RT. We have developed a biomarker model based on CA19-9 and ALBI grade at presentation that can distinguish sub-populations with markedly diverse prognoses. This model can aid the clinical management of this challenging disease.

**Supplementary Information:**

The online version contains supplementary material available at 10.1186/s13014-024-02481-y.

## Background

BTC are rare and aggressive malignancies that originate from the epithelial cells of the gallbladder, intrahepatic, and extrahepatic bile ducts. These cancers are uncommon in the Western world, with an annual rate ranging from 0.35 to 2 cases per 100,000 people; in contrast, in China and Thailand, the incidence can be 40 times higher [1].

BTC often carries a bleak prognosis. While surgery is the only curative treatment, these tumors are usually asymptomatic due to their location, and 60–70% are diagnosed at an advanced stage when surgery is no longer viable [2]. Treatment options for advanced disease patients are limited, with systemic chemotherapy commonly used. Based on the results of the ABC-02 trial, the combination of gemcitabine and cisplatin (GemCis) became a common first-line treatment for advanced BTC, with a demonstrated median OS of 11.7 months [3]. Recent phase III trials showed an improvement in OS of a little over a month with the addition of immunotherapy to GemCis, and this combination has become the new frontline standard [4, 5]. The NCCN guidelines recommend 5-fluorouracil and oxaliplatin (FOLFOX) as a second-line therapy based on the results of the ABC-06 trial. Patients who received FOLFOX had an average OS of 6.2 months, while those in the active symptom control group had an average OS of 5.3 months [6].

RT is an alternative treatment option for patients with unresectable BTC, however its role is less clear due to the lack of data to define a standard regimen or definitive clear survival benefit. Several studies, mainly conducted at single institutions, reported outcomes of RT in unresectable BTC, with or without concurrent chemotherapy. These reports describe different dosing schedules with varying RT doses and a wide range of survival outcomes, from as low as six months to as high as 24 months [7–15]. The pursuit of effective treatment strategies is further complicated by the potential side effects of RT and the protracted treatment schedules, frequently entailing weeks of treatment coupled with concurrent chemotherapy.

Consequently, there is a great need to identify subgroups of patients who can benefit substantially from an intensive therapeutic approach and others with dismal prognoses where burdensome, toxic, and likely ineffective treatments should be avoided. Herein, we sought to identify prognostic markers of survival after RT that could assist in clinical management.

## Methods

### Study design and patient selection

This retrospective cohort study was conducted to comprehensively analyze the outcomes of unresectable BTC patients treated with RT. Patients with non-metastatic BTC treated at the University of Pennsylvania with RT between September 2008 and November 2022 were identified from the electronic medical records. Clinical data were extracted following approval of the institutional review board. Patients were included in this study if the target received a Biologically Effective Dose (BED_10_) of at least 60 Gy. This threshold, biologically equivalent to a dose of 50 Gy in 2 Gy fractions (EQD2), was chosen because, based on our institutional practice, it would exclude patients treated with palliative intent.

### Data collection and variables

Demographic, clinical, and treatment-related data were systematically extracted. Variables included age at presentation, gender, ethnicity, BMI, type of pathology, microscopy pathology, Eastern Cooperative Oncology Group (ECOG) performance status, the presence of known risk factors for BTC, liver cirrhosis, ascites, encephalopathy, ALBI grade at diagnosis, CA19-9 blood levels at diagnosis (before initiating treatment and after initial decompression of the bile duct system in cases of biliary obstruction), biliary cancer type, tumor maximum diameter at diagnosis, T stage at diagnosis, N stage at diagnosis, clinical stage at diagnosis, location of involved lymph nodes at diagnosis, vascular involvement at diagnosis, CEA blood levels at diagnosis, albumin blood levels at diagnosis and total bilirubin blood levels at diagnosis. Treatment details included chemotherapy regimens, RT modality, and treatment planning/dosimetric parameters, including BED_10_ and gross tumor volume (GTV).

### Clinical outcomes and follow-up

Clinical outcomes were documented, including local recurrence, distant metastasis, and mortality during the follow-up period. During the initial presentation, all patients were thoroughly discussed at a multidisciplinary gastrointestinal or hepatobiliary tumor board. Unresectability was determined by experienced surgical oncologists and hepatobiliary surgeons. Treatment-related toxicity was reported during RT using Common Terminology Criteria for Adverse Events CTCAE v4.0 [16]. OS was defined as the time from the last RT treatment to death or last documented follow-up. Patients were followed up with imaging every three months per the National Comprehensive Cancer Network (NCCN) guidelines [17]. This consisted most commonly of dynamic contrast-enhanced MRI, contrast-enhanced computed tomography (CT), or positron emission tomography (PET). All scans were read by specialized gastrointestinal radiologists to determine the pattern of first recurrence. Local progression was defined as the progression of the tumor in the irradiated field. Follow-up time was calculated from the end of the RT course. Patterns of failure were assessed, and causes of death were identified through medical record review. Patients without recurrence or death were censored at the last follow-up.

### Statistical analysis

Variables were compared using a two-tailed Student’s T-test for continuous variables and a Chi-square for categorical variables. A univariate and multivariate Cox proportional hazard analysis was conducted to identify parameters associated with OS following RT with variables related to patient demographics, clinical characteristics, and treatment variables. Local recurrence-free survival, distant metastasis-free survival, and OS were calculated using the Kaplan-Meier method. Survival outcome differences were evaluated using the log-rank test [18]. Statistical analysis was performed using SPSS version 28 (SPSS, Inc., Chicago, Illinois, USA). For all calculations, *P* values < 0.05 were considered statistically significant.

## Results

### Patient characteristics and demographics

The analytic cohort included seventy-eight patients (Table 1). Most were white males with good performance status (91.8% ECOG 0–1), without known risk factors for BTC. The majority of patients did not have cirrhosis, ascites, or encephalopathy at presentation; most had an ALBI grade of 2. The median CA19-9 blood level at diagnosis (before initiating any oncologic treatment and after biliary decompression in cases of biliary obstruction) was 63 U/ml. The most common type of BTC was intrahepatic, followed by hilar and extrahepatic. Four patients were diagnosed with gallbladder cancer, and a similar number with intrahepatic and hepatocellular carcinoma (HCC). Over a third of the tumors presented with regional nodal disease, most commonly portocaval. Nearly half presented with a locally advanced stage, and a quarter of the patients had vascular involvement when they were diagnosed.

**Table 1:** Patient characteristics of the entire cohort

		All patients *N* = 78 (%)
Age (mean ± SD, years)		71.15 ± 10.46
Gender male		41 (52.6)
Ethnicity	White	70 (89.7)
	Non-white	8 (10.3)
BMI		27.5 ± 5.7
Type of pathology	Cytology	8 (10.4)
	Biopsy	69 (89.6)
Microscopy pathology	Adenocarcinoma	62 (79.5)
	Non adenocarcinoma	8 (10.3)
	Unknown	8 (10.3)
ECOG	0	26 (35.6)
	1	41 (56.2)
	2	4 (5.5)
	3	2 (2.7)
Risk factors	Hepatitis B	1 (1.3)
	Hepatitis C	9 (11.8)
	Primary sclerosing cholangitis	3 (3.9)
	Cirrhosis	7 (9.2)
	Anatomic anomaly	1 (1.3)
	Alcohol	4 (5.3)
	Unknown	53 (67.9)
Ascites at presentation	Absent	63 (87.2)
	Mild	6 (7.7)
	Moderate	3 (3.8)
Encephalopathy grade at presentation	None	75 (96.2)
	Minimal (grade 1, 2)	2 (2.6)
	Advanced (grade 3, 4)	0 (0)
Cirrhosis at presentation	Yes	20 (25.6)
Type of biliary cancer	Intrahepatic	41 (52.6)
	Hillar	15 (19.2)
	Extrahepatic	13 (16.7)
	Gallbladder	4 (5.1)
	Intrahepatic and HCC	4 (5.1)
	Unknown	1 (1.3)
Type of diagnosis	Primary	74 (96.1)
	Recurrence	3 (3.9)
Tumor maximum diameter (median ± SD, cm)		3.4 ± 3.21
T stage	T0	1 (1.3)
	T1	29 (37.2)
	T2	24 (30.8)
	T3	13 (16.7)
	T4	8 (10.3)
	Tx	2 (2.6)
	Recurrence	1 (1.3)
N stage	0	47 (60.3)
	1	30 (38.5)
	2	1 (1.3)
Overall stage	I	24 (30.8)
	II	12 (15.4)
	III	36 (46.2)
	IV	5 (6.4)
	Recurrence	1 (1.3)
Location of involved lymph nodes	Portocaval	29 (37.2)
	Cystic	0 (0)
	Retroduedenal	1 (1.3)
	Paraaortic	4 (5.1)
	Superior mesenteric artery	1 (1.3)
	Celiac trunk	2 (2.6)
	Gastrohepatic	3 (3.8)
	Other	5 (6.4)
Vascular involvement		20 (25.6)
CA19-9 at diagnosis (median, Q1, Q3 U/ml)		63.0, 33.0, 215.6
CEA at diagnosis (median, Q1, Q3 ng/ml)		2.4, 1.8, 3.7
Albumin (median, Q1, Q3 g/dL)		3.7, 3.4, 4.1
Bilirubin (median, Q1, Q3 mg/dL)		0.9, 0.7, 1.3
ALBI grade	1	26 (33.3)
	2	45 (57.7)
	3	4 (5.1)

### Treatment details for the entire cohort

Table 2 details the oncologic treatment of the entire cohort. Most patients received a hypofractionated RT regimen (2.01–5 Gy per fraction) and concurrent 5FU-based chemotherapy (either 5FU or capecitabine). The median BED_10_ was 73.1 Gy. A similar number of patients received proton therapy compared to photon-based RT. Approximately half of the patients received chemotherapy either before or after RT. Among the 37 patients who did not receive systemic therapy, over half were unable to do so due to poor performance status and comorbidities (Supplementary Table 1). In 18.9% of these cases, the medical oncologist recommended systemic chemotherapy, but the patients declined. Of the 41 patients who did receive systemic treatment, most underwent chemotherapy prior to starting RT (Supplementary Table 2). The most common regimen was a combination of GemCis (Supplementary Table 3). Only a small minority of patients received second and third-line systemic therapies.

**Table 2:** Radiation treatment details of the entire cohort

		All patients *N* = 78 (%)
Dose per fraction	Conventional (180–200 cGY/Fx)	33 (42.3)
	Hypofractionated (201–500 cGY/Fx)	42 (53.8)
	Ultrafractionated (≥ 501 cGY/Fx)	3 (3.8)
Number of fractions	1–5	4 (5.1)
	6–20	26 (33.3)
	≥ 21	48 (61.5)
BED_10_ (median, Q1, Q3, Gy)		73.1, 67.6, 81.2
Treatment gap	Yes	11 (14.1)
RT treatment modality	Proton	32 (41.0)
	Photons	37 (47.4)
	Proton and photons	8 (10.3)
Chemotherapy concurrent	Yes	48 (61.5)
Type concurrent chemotherapy	Capecitabine	36 (75.0)
	5FU	11 (22.9)
	Other	1 (2.9)
Chemotherapy other than concurrent	None	37 (47.4)
	Before and/or after RT	41 (52.6)
GTV volume (median, Q1, Q3 cm³)		64.3, 33.3, 150.4

### Clinical outcomes and patterns of failure

Of the 78 patients in the study, five patients (6.4%) had documented grade (G)3 and above gastrointestinal toxicity (three patients with G3, one G4, and one died from RT-induced enteritis), four patients (5.1%) experienced G3 fatigue, two patients (2.6%) had G3 abdominal pain and non-had G3 and above skin toxicity.

Fifty-nine (77.6%) died during the follow-up period, with a median OS of 12.3 months after RT (Supplementary Fig. 1); twenty (27.0%) had a local recurrence in the irradiated field as the first site of failure, and thirty-seven (47.4%) developed distant metastasis (Table 3). The most common sites of metastatic spread following RT were the peritoneum and liver, while the most common cause of death was liver failure followed by biliary sepsis. In patients with a local recurrence, the median time to recurrence was 30.1 months (Supplementary Fig. 2), while the median metastasis-free survival was 11.0 months (Supplementary Fig. 3). In patients who developed distant metastasis, the median time to death after metastatic disease was 4.9 months, while in patients with a local recurrence, the median time to death after diagnosis of progression at the RT site was 5.1 months.

**Table 3:** Clinical outcomes and patterns of failure

		All patients *N* = 78 (%)
Distant metastasis		37 (47.4)
	Peritoneal carcinomatosis	10 (27.0)
	Liver metastasis	10 (27.0)
	Lung and liver metastasis	4 (10.8)
	Distant lymph node metastasis	2 (5.4)
	Lung metastasis	2 (5.4)
	Other	9 (24.3)
Local recurrence		20 (27.0)
Death		59 (77.6)
	Liver failure	18 (30.5)
	Biliary sepsis	17 (28.8)
	Distant disease	8 (13.5)
	Gastrointestinal bleeding	4 (6.7)
	Other	5 (8.4)
	Unknown	7 (11.8)
Follow-up time (months)		12.03

### Univariate and multivariate Cox regression analysis for predictors of OS after RT

Table 4 shows the results of a univariate Cox proportional hazard analysis for OS after RT using the patient’s age (above vs. under 70 years), gender, ECOG performance status, ALBI grade, type of biliary cancer (intrahepatic vs. hilar vs. extrahepatic vs. gallbladder) vascular involvement, clinical stage, GTV volume (above vs. under median value of 64.3 cm³), CEA blood levels at diagnosis (above vs. under median value of 2.4 ng/ml), CA19-9 blood levels at diagnosis (above vs. under median value of 63 U/ml), RT modality (protons vs. photons vs. mixed), BED_10_ (above vs. under median 73.1 Gy), concurrent chemotherapy during RT and systemic chemotherapy other than concurrent (before or after RT). Parameters that reached a *P* < 0.1 level of statistical significance in the univariate analysis were selected for inclusion in a multivariate Cox proportional hazard analysis. In addition, given previous studies demonstrating an association between clinical stage and treatment outcomes in BTC patients, we included clinical stage in the multivariate analysis [17, 19]. In the multivariate Cox regression, CA19-9 above the median value was a significant predictor of OS with a hazard ratio (HR) of 2.621 (*P* = 0.003). In addition, a higher ALBI grade was also associated with a statistically significant decreased OS after RT (HR = 1.952, *P* = 0.021).

**Table 4:** Univariate and multivariate Cox regression analysis for OS after RT

		Number at risk	Cumulative probability of death %	Univariate analysis				Multivariate analysis			
				HR	95.0% CI		*P* value	HR	95.0% CI		*P* value
					Lower	Upper			Lower	Upper	
Age	> 70 years old vs. < 70 years old	31	72.1	0.751	0.449	1.257	0.277				
Gender	male vs. female	32	82.1	1.135	0.678	1.900	0.631				
ECOG		55	77.5	1.369	0.993	1.886	0.055	1.030	0.683	1.554	0.887
Type of biliary cancer	Intrahepatic with/without HCC	43	67.4	Reference				Reference			
	Hillar	15	86.7	1.131	0.585	2.187	0.713	0.735	0.331	1.630	0.448
	Extrahepatic	13	100	1.939	1.001	3.757	0.050	0.796	0.339	1.866	0.600
	Gall bladder	4	75	2.218	0.666	7.384	0.194	6.904	0.619	77.057	0.116
Vascular involvement		24	91.7	1.272	0.741	2.184	0.383				
Overall stage		76	77.6	1.171	0.903	1.518	0.235	1.058	0.772	1.450	0.725
CA19-9 at diagnosis	> 63 U/ml vs. ≤ 63 U/ml	35	88.6	3.054	1.733	5.382	0.001>	2.621	1.390	4.944	0.003
CEA at diagnosis	> 2.4 ng/ml vs. ≤ 2.4 ng/ml	23	82.6	1.262	0.666	2.395	0.476				
ALBI grade		73	79.5	2.077	1.259	3.427	0.004	1.952	1.106	3.443	0.021
GTV volume	> 64.3 cm³ vs. ≤ 64.3 cm³	28	67.9	0.874	0.482	1.586	0.658				
BED_10_	> 73.1 Gy vs. ≤ 73.1 Gy	38	73.7	0.694	0.414	1.163	0.166				
RT treatment modality	Proton vs. photon vs. proton and photons	75	77.3	0.808	0.480	1.362	0.424				
Chemotherapy concurrent	Yes	46	84.8	1.155	0.673	1.982	0.602				
Chemotherapy other than concurrent	Yes	41	85.0	0.961	0.566	1.632	0.884				

### Impact of CA19-9 blood levels at the presentation on clinical outcomes after RT

**Fig. 1:**
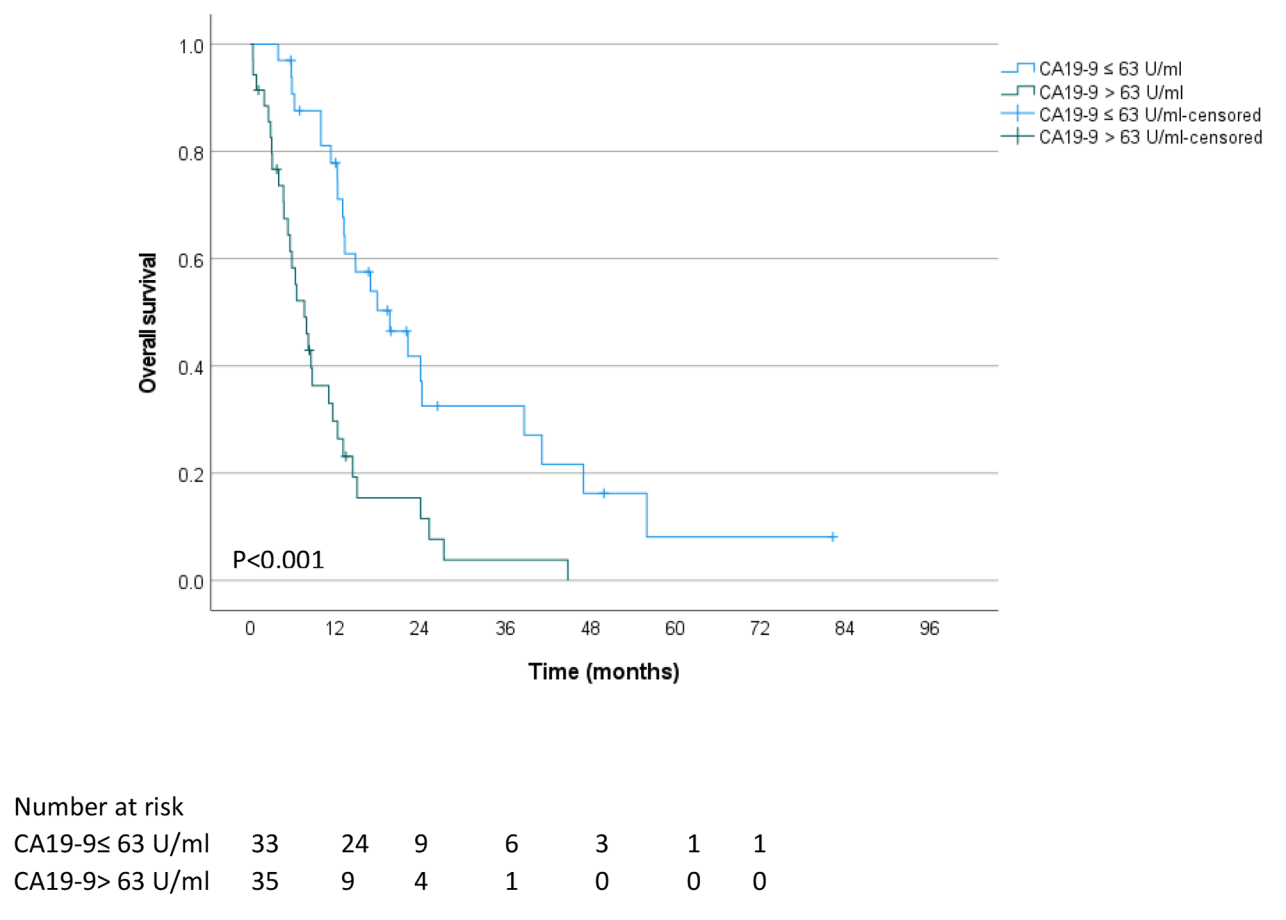
Overall survival of unresectable BTC patients who received RT were stratified based on their plasma CA19-9 levels at presentation, above and below the median of 63 U/ml

**Fig. 2:**
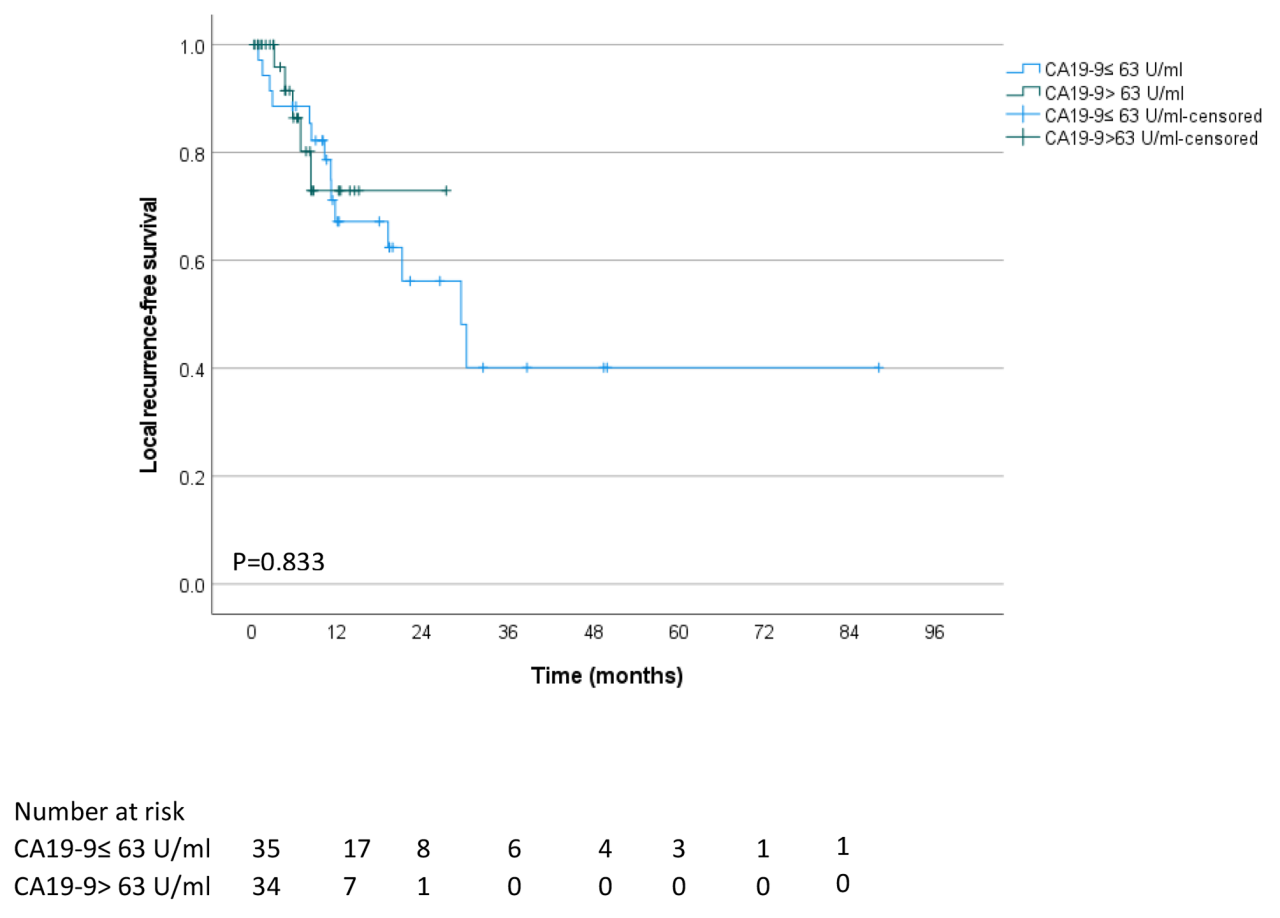
Local recurrence-free survival of unresectable BTC patients who received RT were stratified based on their plasma CA19-9 levels at presentation, above and below the median of 63 U/ml

**Fig. 3:**
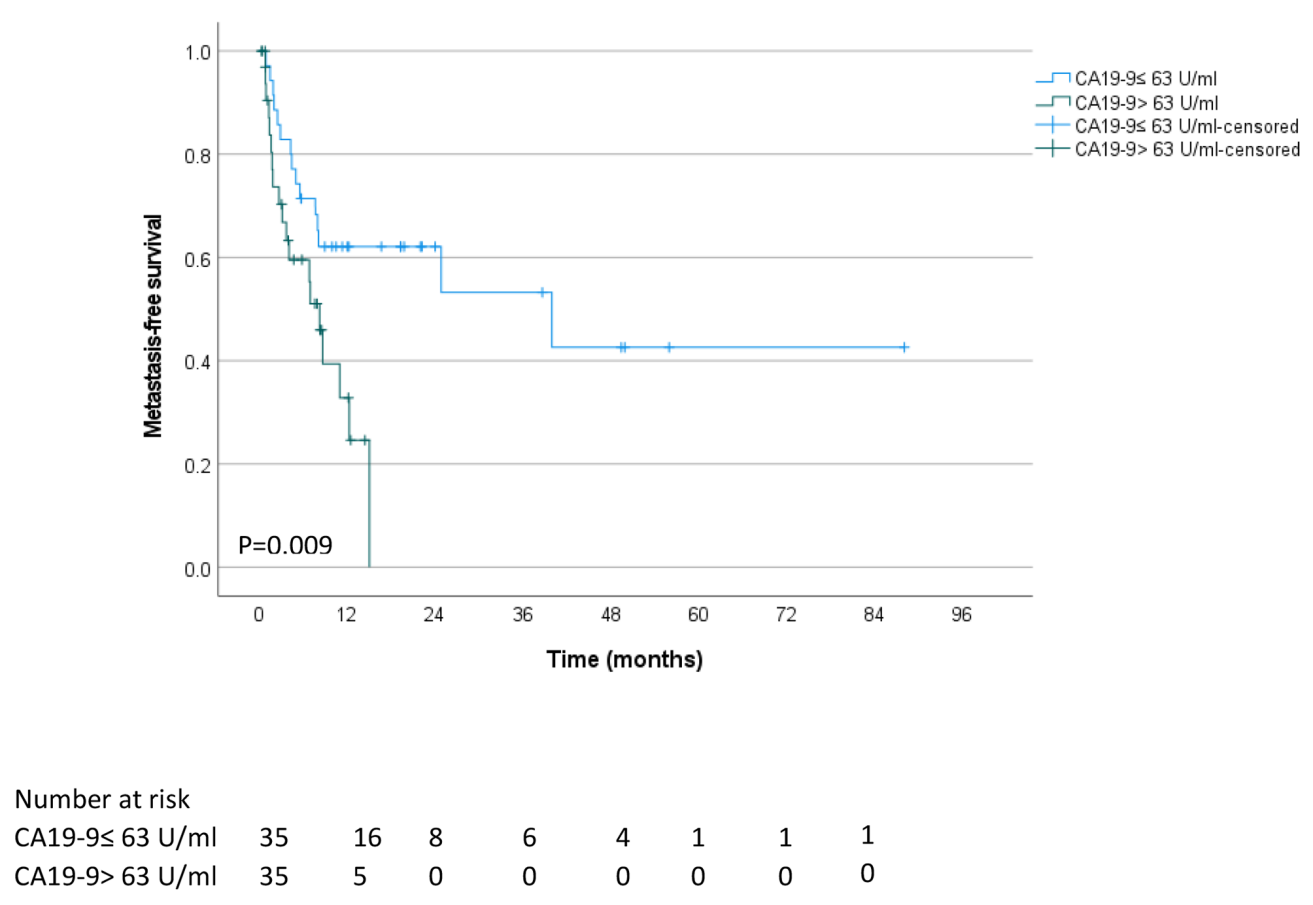
Metastasis-free survival of unresectable BTC patients who received RT were stratified based on their plasma CA19-9 levels at presentation, above and below the median of 63 U/ml

Supplementary Table 4 compares the characteristics of patients with CA19-9 blood levels at presentation over and under the median value of ≤ 63 U/ml. Patients with high CA19-9 blood levels had comparable mean age, ethnicity, ECOG performance status, and prevalence of encephalopathy, ascites, and cirrhosis at diagnosis. In addition, there was no difference in the prevalence of vascular involvement as assessed by imaging scans or tumor diameter. However, compared to patients with low CA19-9 blood levels, patients with a high biomarker level had a higher clinical stage, with 60.0% diagnosed with a stage III disease compared to 28.6%, and had significantly worse ALBI grade. There was no clinically significant difference in the RT regimens, i.e., BED_10_, number of fractions or dose per fraction, concurrent chemotherapy, and systemic therapy (Supplementary Table 5). However, the outcome of patients with high CA19-9 at presentation treated with RT was dismal, with a median survival of 7.6 months after RT compared with 19.7 months in patients with low CA19-9 levels (*P* < 0.001, Fig. 1). There was no difference in local recurrence-free survival between patients with high and low CA19-9 blood levels at presentation (*P* = 0.833, Fig. 2); however, patients with high CA19-9 had shorter metastasis-free survival (*P* = 0.009, Fig. 3).

### Combining CA19-9 blood levels at presentation and ALBI grade to predict survival after RT in unresectable BTC patients

**Fig. 4:**
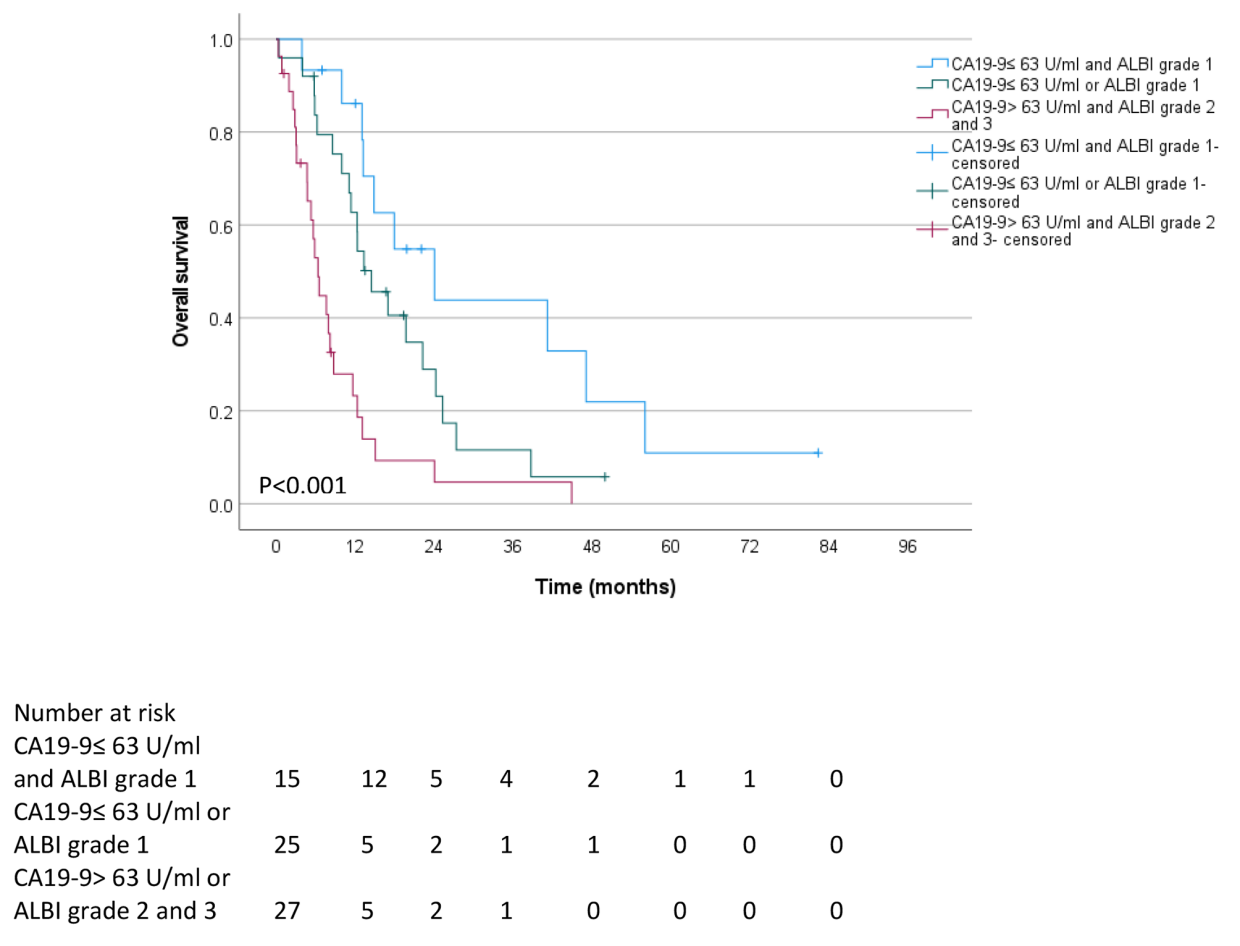
A model combining CA19-9 blood levels at presentation and ALBI grade to predict overall survival after RT in unresectable BTC patients

CA19-9 blood levels at presentation and ALBI grade at baseline were significant predictors of poor survival after RT in our multivariate Cox proportional hazard model in patients with unresectable BTC. To further identify a subgroup of patients who may benefit from RT as opposed to patients where RT should be avoided, we created a new variable combining CA19-9 and ALBI grade (Fig. 4). Patients with CA19-9 blood levels at presentation under (or equal) the median value of 63 U/ml and ALBI grade 1 at baseline had a median survival of 24.0 months after RT. Patients with CA19-9 blood levels at presentation over the median value of 63 U/ml and ALBI grade 2 or 3 at baseline had a median survival of 6.3 months after RT. All other patients (i.e., patients with CA19-9 blood levels at presentation under (or equal) the median value of 63 U/ml or ALBI grade 1 at baseline) had a median survival of 14.4 months after RT.

## Discussion

This study provides a comprehensive analysis of patient characteristics, treatment modalities, clinical outcomes, and failure patterns in a relatively large cohort of unresectable BTC patients treated with RT with definitive intent at the University of Pennsylvania. It demonstrates that CA-19-9 and ALBI grade at diagnosis are statistically and clinically significant predictors of survival after RT and that the biomarker model we have developed can define subgroups of patients with very contrasting outcomes. We submit that this risk stratification can help guide clinical decision-making.

The cohort in this study predominantly comprised white patients with good performance status and lacked known risk factors for BTC. This distribution aligns with the general demographics observed in cholangiocarcinoma populations, often showing a preference for males and a higher incidence in Caucasians [20–23]. The majority of patients presented without cirrhosis, ascites, or encephalopathy, reflecting the cohort’s relatively favorable baseline health.

In this study, we opted to use the ALBI grade to assess patients’ baseline liver function. Recent studies have shown that the ALBI grade performs better than the Child-Pugh grade in evaluating liver function, complications, and prognosis in HCC [24–28], intrahepatic cholangiocarcinoma [29, 30] and extrahepatic cholangiocarcinoma [31]. We found that ALBI grade was a strong predictor of OS, second only to CA19-9. Interestingly, both ALBI grade and CA19-9 (each on their own) outperformed the more conventional predictors such as T stage and N stage. This suggests that each is a surrogate of tumor burden, likely more accurate than the TNM stage for BTC cancer patients undergoing RT. In addition to being a surrogate of tumor volume, ALBI grade also reflects baseline liver function related to any preexisting liver disease as well as the physiological impact of the newly diagnosed BTC. These, undoubtedly, have a significant impact on a patient’s ability to tolerate treatment.

Our treatment approach primarily involved a hypofractionated RT regimen and concurrent 5FU-based chemotherapy. This treatment modality aligns with current standards for unresectable BTC [32–35]. Approximately half of the patients received chemotherapy before or after RT, indicating variability in treatment sequences. Additionally, an equal proportion of patients received proton therapy compared to photon-based RT, showcasing the evolving landscape of RT modalities in cancer care.

The clinical outcomes observed in this study reveal challenges in managing unresectable BTC. A substantial proportion of patients developed distant metastasis, and the mortality rate was high. The median overall survival after RT of 12.3 months underscores the aggressive nature of this disease. The patterns of metastatic spread, with the peritoneum and liver being common sites, align with the typical behavior of BTC [31, 36]. Liver failure emerged as the primary cause of death, emphasizing the critical role of hepatic function in patient survival.

It is noteworthy that in our study, the addition of systemic therapy was not associated with prolonged survival in patients undergoing RT. A significant limitation is that none of the patients received durvalumab, which has recently demonstrated improved survival outcomes in patients with unresectable, recurrent, or metastatic BTC combined with GemCis [5]. One possible explanation for our findings is that in unresectable BTC, death is often due to liver failure, as demonstrated in our study, highlighting the crucial importance of RT in achieving local control. The potential benefits of chemotherapy beyond those provided by local therapy alone remain uncertain.

Notably, the univariate and multivariate Cox regression analyses identified blood CA19-9 levels above the median of 63 U/ml and a higher ALBI grade at presentation as significant predictors of poor survival after RT. Combining CA19-9 and ALBI grades at presentation further refined survival prediction. Patients with CA19-9 blood levels at the time of presentation that were equal to or below the median value of 63 U/ml and ALBI grade 1 at baseline survived for a median time of 24.0 months after undergoing RT. On the other hand, patients who had CA19-9 blood levels at presentation greater than the median value of 63 U/ml and ALBI grade 2 or 3 at baseline had a median survival time of 6.3 months after undergoing RT.

Recognizing these stark differences in survival offers an opportunity for a more tailored approach in unresectable BTC patients. For instance, one could consider a more aggressive staging workup that includes routine use of a PET/CT in patients at high risk or a more protracted period of chemotherapy before RT (or chemoRT) in these patients. In the worst prognostic subgroup we have identified, those with both a high CA19-9 and a high ALBI grade, one could consider a more palliative approach. Future research should explore the potential impact of incorporating these biomarkers into treatment algorithms and investigate strategies to improve outcomes for high-risk patient subgroups. These findings could potentially be helpful in designing future clinical trials by using them as stratification factors. This approach could improve the quality of the trials and increase their chances of success. All of this could contribute to better patient outcomes in a rare disease where quality data is scarce.

## Conclusions

This study provides valuable insights into the heterogeneity of outcomes in unresectable BTC patients undergoing RT. The findings underscore the importance of individualized risk assessment and highlight the potential of integrating readily available biomarkers, CA19-9 blood levels and ALBI grade, into treatment decision-making in this challenging patient population.

### Electronic supplementary material

Below is the link to the electronic supplementary material.

Supplementary Material 1

Supplementary Material 2

Supplementary Material 3

Supplementary Material 4

## Data Availability

Data is not available for reuse. Please contact the author for data requests.

## Abbreviations

5FU: Fluorouracil
ALBI: Albumin-bilirubin prognostic score
BED: Biologically effective dose
CA19: 9-Carbohydrate antigen 19 − 9
CEA: Carcinoembryonic antigen
ECOG: Eastern Cooperative Oncology Group
EHR: Electronic health record
Fx: Fraction
GTV: Gross tumor volume
HCC: Hepatocellular carcinoma
NCCN: National Comprehensive Cancer Network
RT: Radiation Therapy

## References

[1] Bridgewater JA, Goodman KA, Kalyan A, et al. Biliary tract cancer: Epidemiology, radiotherapy, and molecular profiling. Am Soc Clin Oncol Educational book Am Soc Clin Oncol Annual Meeting. 2016;35:e194–203. 10.1200/EDBK_160831 27249723

[2] Tella SH, Kommalapati A, Borad MJ, et al. Second-line therapies in advanced biliary tract cancers. Lancet Oncol. 2020;21:e29–41. 31908303 10.1016/S1470-2045(19)30733-8

[3] Valle J, Wasan H, Palmer DH, et al. Cisplatin plus gemcitabine versus gemcitabine for biliary tract cancer. N Engl J Med. 2010;362:1273–81. 20375404 10.1056/NEJMoa0908721

[4] Kelley RK, Ueno M, Yoo C, et al. Pembrolizumab in combination with gemcitabine and cisplatin compared with gemcitabine and cisplatin alone for patients with advanced biliary tract cancer (keynote-966): a randomised, double-blind, placebo-controlled, phase 3 trial. Lancet (London England). 2023;401:1853–65. 37075781 10.1016/S0140-6736(23)00727-4

[5] Oh D-Y, He AR, Qin S, et al. Durvalumab plus gemcitabine and cisplatin in advanced biliary tract cancer. NEJM Evid. 2022;1:EVIDoa2200015. 38319896 10.1056/EVIDoa2200015

[6] Lamarca A, Palmer DH, Wasan HS, et al. Second-line folfox chemotherapy versus active symptom control for advanced biliary tract cancer (abc-06): a phase 3, open-label, randomised, controlled trial. Lancet Oncol. 2021;22:690–701. 33798493 10.1016/S1470-2045(21)00027-9 PMC8082275

[7] Foo ML, Gunderson LL, Bender CE, et al. External radiation therapy and transcatheter iridium in the treatment of extrahepatic bile duct carcinoma. Int J Radiat Oncol Biol Phys. 1997;39:929–35. 9369143 10.1016/S0360-3016(97)00299-X

[8] Lee KJ, Yi SW, Cha J, et al. A pilot study of concurrent chemoradiotherapy with gemcitabine and cisplatin in patients with locally advanced biliary tract cancer. Cancer Chemother Pharmacol. 2016;78:841–6. 27586966 10.1007/s00280-016-3143-2

[9] Moureau-Zabotto L, Turrini O, Resbeut M, et al. Impact of radiotherapy in the management of locally advanced extrahepatic cholangiocarcinoma. BMC Cancer. 2013;13:568. 24299517 10.1186/1471-2407-13-568 PMC4219485

[10] Crane CH, Macdonald KO, Vauthey JN, et al. Limitations of conventional doses of chemoradiation for unresectable biliary cancer. Int J Radiat Oncol Biol Phys. 2002;53:969–74. 12095564 10.1016/S0360-3016(02)02845-6

[11] Bisello S, Buwenge M, Palloni A, et al. Radiotherapy or chemoradiation in unresectable biliary cancer: a retrospective study. Anticancer Res. 2019;39:3095–100. 31177154 10.21873/anticanres.13445

[12] Brunner TB, Schwab D, Meyer T, et al. Chemoradiation may prolong survival of patients with non-bulky unresectable extrahepatic biliary carcinoma. A retrospective analysis. Strahlentherapie Und Onkologie: Organ Der Deutschen Rontgengesellschaft [et al]. 2004;180:751–7. 10.1007/s00066-004-1315-1 15592694

[13] Ben-David MA, Griffith KA, Abu-Isa E, et al. External-beam radiotherapy for localized extrahepatic cholangiocarcinoma. Int J Radiat Oncol Biol Phys. 2006;66:772–9. 17011452 10.1016/j.ijrobp.2006.05.061

[14] Elganainy D, Holliday EB, Taniguchi CM, et al. Dose escalation of radiotherapy in unresectable extrahepatic cholangiocarcinoma. Cancer Med. 2018;7:4880–92. 30152073 10.1002/cam4.1734 PMC6198206

[15] Chen SC, Chen MH, Li CP, et al. External beam radiation therapy with or without concurrent chemotherapy for patients with unresectable locally advanced hilar cholangiocarcinoma. Hepatogastroenterology. 2015;62:102–7. 25911877

[16] NCI. National cancer institute common terminology criteria for adverse events (CTCAE), Version 4.0. 2010. https://ctep.cancer.gov/protocolDevelopment/electronic_applications/ctc.htm. Accessed 17 May 2010.

[17] Dhanasekaran R, Hemming AW, Zendejas I, et al. Treatment outcomes and prognostic factors of intrahepatic cholangiocarcinoma. Oncol Rep. 2013;29:1259–67. 23426976 10.3892/or.2013.2290 PMC3621732

[18] Goel MK, Khanna P, Kishore J. Understanding survival analysis: Kaplan-Meier estimate. Int J Ayurveda Res. 2010;1:274–8. 21455458 10.4103/0974-7788.76794 PMC3059453

[19] Doussot A, Groot-Koerkamp B, Wiggers JK, et al. Outcomes after resection of intrahepatic cholangiocarcinoma: external validation and comparison of prognostic models. J Am Coll Surg. 2015;221:452–61. 26206643 10.1016/j.jamcollsurg.2015.04.009 PMC4784264

[20] Brindley PJ, Bachini M, Ilyas SI, et al. Cholangiocarcinoma. Nat Reviews Disease Primers. 2021;7:65. 34504109 10.1038/s41572-021-00300-2 PMC9246479

[21] Mosadeghi S, Liu B, Bhuket T, et al. Sex-specific and race/ethnicity-specific disparities in cholangiocarcinoma incidence and prevalence in the USA: an updated analysis of the 2000–2011 surveillance, epidemiology and end results registry. Hepatol Research: Official J Japan Soc Hepatol. 2016;46:669–77. 10.1111/hepr.12605 26508039

[22] Tyson GL, El-Serag HB. Risk factors for cholangiocarcinoma. Hepatology (Baltimore MD). 2011;54:173–84. 21488076 10.1002/hep.24351 PMC3125451

[23] Mukkamalla SKR, Naseri HM, Kim BM, et al. Trends in incidence and factors affecting survival of patients with cholangiocarcinoma in the United States. J Natl Compr Cancer Network: JNCCN. 2018;16:370–6. 10.6004/jnccn.2017.7056 29632056

[24] Chan AW, Chong CC, Mo FK, et al. Applicability of albumin-bilirubin-based Japan integrated staging score in hepatitis b-associated hepatocellular carcinoma. J Gastroenterol Hepatol. 2016;31:1766–72. 26992142 10.1111/jgh.13339

[25] Wang YY, Zhong JH, Su ZY, et al. Albumin-bilirubin versus child-pugh score as a predictor of outcome after liver resection for hepatocellular carcinoma. Br J Surg. 2016;103:725–34. 27005482 10.1002/bjs.10095

[26] Zhao S, Wang M, Yang Z, et al. Comparison between child-pugh score and albumin-bilirubin grade in the prognosis of patients with hcc after liver resection using time-dependent Roc. Annals Translational Med. 2020;8:539. 10.21037/atm.2020.02.85 PMC7214905 32411762

[27] Su T-S, Yang H-M, Zhou Y, et al. Albumin - bilirubin (albi) versus child-turcotte-pugh (ctp) in prognosis of hcc after stereotactic body radiation therapy. Radiat Oncol. 2019;14:50. 30917853 10.1186/s13014-019-1251-y PMC6436219

[28] Toyoda H, Johnson PJ. The albi score: from liver function in patients with hcc to a general measure of liver function. JHEP Reports: Innov Hepatol. 2022;4:100557. 10.1016/j.jhepr.2022.100557 PMC9482109 36124124

[29] Kaneko S, Kurosaki M, Tsuchiya K, et al. Prognosis of intrahepatic cholangiocarcinoma stratified by albumin-bilirubin grade. Hepatol Research: Official J Japan Soc Hepatol. 2021;51:902–8. 10.1111/hepr.13673 34046984

[30] Tsilimigras DI, Hyer JM, Moris D, et al. Prognostic utility of albumin-bilirubin grade for short- and long-term outcomes following hepatic resection for intrahepatic cholangiocarcinoma: a multi-institutional analysis of 706 patients. J Surg Oncol. 2019;120:206–13. 31025380 10.1002/jso.25486

[31] Wang Y, Pang Q, Jin H, et al. Albumin-bilirubin grade as a novel predictor of survival in advanced extrahepatic cholangiocarcinoma. Gastroenterol Res Pract. 2018;2018:8902146. 30622562 10.1155/2018/8902146 PMC6304808

[32] Wang N, Huang A, Kuang B, et al. Progress in radiotherapy for cholangiocarcinoma. Front Oncol. 2022;12:868034. 35515132 10.3389/fonc.2022.868034 PMC9063097

[33] Rizvi S, Khan SA, Hallemeier CL, et al. Cholangiocarcinoma - evolving concepts and therapeutic strategies. Nat Reviews Clin Oncol. 2018;15:95–111. 10.1038/nrclinonc.2017.157 PMC5819599 28994423

[34] Koo T, Park HJ, Kim K. Radiation therapy for extrahepatic bile duct cancer: current evidences and future perspectives. World J Clin Cases. 2019;7:1242–52. 31236388 10.12998/wjcc.v7.i11.1242 PMC6580339

[35] Network NCC. Biliary tract cancers. Version: 3.2023. In: Editor, editor^editors. Book Biliary tract cancers. Version: 3.2023.; 2023.

[36] Razumilava N, Gores GJ. Cholangiocarcinoma. Lancet (London England). 2014;383:2168–79. 24581682 10.1016/S0140-6736(13)61903-0 PMC4069226

