# Supplementary material for: Clinical outcomes and risk stratification in unresectable biliary tract cancers undergoing radiation therapy
Source: Radiat Oncol. 2024 Aug 1;19:102. doi: 10.1186/s13014-024-02481-y (PMC11293151; doi:10.1186/s13014-024-02481-y)
Supplement: Supplementary file 1 — Supplementary Material 1 [file 13014_2024_2481_MOESM1_ESM.docx]

| **Supplementary Table 1. Reasons for not receiving systemic therapy N=37 (%)** | | |  |  |
| --- | --- | --- | --- | --- |
| Patient’s preference | 7 (18.9) |  |  |  |
| Comorbidities/poor performance status | 20 (54.0) |  |  |  |
| Stable disease after RT | 4 (10.8) |  |  |  |
| Admitted to hospice/death | 1 (2.7) |  |  |  |
| Unknown | 5 (13.5) |  |  |  |

**Supplementary Table 2. Sequence of systemic therapy N=41 (%)**

| Chemotherapy before RT | 28 (68.2) |
| --- | --- |
| Chemotherapy after RT | 6 (14.6) |
| Chemotherapy before and after RT | 7 (17.0) |

**Supplementary Table 3. Details of systemic therapy regimens N=41 (%)**

| 1st line |  | 41 (100.0) |
| --- | --- | --- |
|  | Gemcitabine monotherapy | 6 (14.6) |
|  | Gemcitabine and oxaliplatin | 5 (12.1) |
|  | Cisplatin and gemcitabine | 23 (56.0) |
|  | Cisplatin, gemcitabine and nab-paclitaxel | 4 (9.7) |
|  | 5-FU, leucovorin and irinotecan | 1 (2.4) |
|  | 5-FU, leucovorin and oxaliplatin | 1 (2.4) |
|  | Cisplatin and etoposide | 1 (2.4) |
| 2nd line |  | 5 (12.2) |
|  | Gemcitabine and nab-paclitaxel | 1 (2.4) |
|  | 5-FU, leucovorin and irinotecan | 2 (2.5) |
|  | 5-FU, leucovorin and oxaliplatin | 1 (2.4) |
|  | Capecitabine monotherapy | 1 (2.4) |
|  | Nivolumab | 1 (2.4) |
| 3rd line |  | 2 (2.5) |
|  | 5-FU, leucovorin and oxaliplatin | 1 (2.4) |
|  | Ivosidenib | 1 (2.4) |

| **Supplementary Table 4. Patient characteristics of low and high CA19-9 blood levels at presentation** | | | | |
| --- | --- | --- | --- | --- |
|  |  | CA19-9 at diagnosis ≤ 63 U/ml, N=35 (%) | CA19-9 at diagnosis > 63 U/ml, N=35 (%) | P value |
| Age (mean± SD, years) |  | 69.37± 10.79 | 72.63± 10.57 | 0.207 |
| Gender male |  | 19 (54.3) | 16 (45.7) | 0.473 |
| Ethnicity | White | 32 (91.4) | 31 (88.6) | 0.690 |
|  | Non-white | 3 (8.6) | 4 (11.4) |  |
| BMI |  | 27.16± 4.57 | 27.92± 7.13 | 0.598 |
| Microscopy pathology | Adenocarcinoma | 26 (89.7) | 30 (90.9) | 0.868 |
|  | Non adenocarcinoma | 3 (10.3) | 3 (9.1) |  |
| ECOG | 0 | 14 (42.4) | 10 (30.3) | 0.356 |
|  | 1 | 17 (51.5) | 20 (60.6) |  |
|  | 2 | 2 (6.1) | 1 (3.0) |  |
|  | 3 | 0 (0) | 2 (6.1) |  |
| Ascites at presentation | Absent | 31 (88.6) | 30 (85.7) | 0.602 |
|  | Mild | 2 (5.7) | 4 (11.4) |  |
|  | Moderate | 2 (5.7) | 1 (2.9) |  |
| Encephalopathy grade at presentation | None | 34 (97.1) | 34 (97.1) | 1 |
|  | Minimal (grade 1, 2) | 1 (2.9) | 1 (2.9) |  |
|  | Advanced (grade 3, 4) | 0 (0) | 0 (0) |  |
| Cirrhosis at presentation | Yes | 8 (22.9) | 10 (29.4) | 0.535 |
| Type of biliary cancer | Intrahepatic | 20 (65.7) | 16 (47.1) | 0.314 |
|  | Hillar | 4 (11.4) | 10 (29.4) |  |
|  | Extrahepatic | 4 (11.4) | 5 (14.7) |  |
|  | Gall bladder | 1 (2.9) | 1 (2.9) |  |
|  | Intrahepatic and HCC | 3 (8.6) | 1 (2.9) |  |
|  | Unknown | 0 (0) | 1 (2.9) |  |
| Tumor maximum diameter (median± SD, cm) |  | 4.9± 3.26 | 4.2± 3.23 | 0.401 |
| T stage | T0 | 0 (0) | 0 (0) | 0.273 |
|  | T1 | 14 (40) | 13 (37.1) |  |
|  | T2 | 13 (37.1) | 9 (25.7) |  |
|  | T3 | 2 (5.7) | 9 (25.7) |  |
|  | T4 | 4 (11.4) | 3 (8.6) |  |
|  | Tx | 1 (2.9) | 1 (2.9) |  |
|  | Recurrence | 1 (2.9) | 0 (0) |  |
| N stage | 0 | 23 (65.7) | 19 (54.3) | 0.316 |
|  | 1 | 11 (31.4) | 16 (45.7) |  |
|  | 2 | 1 (2.9) | 0 (0) |  |
| Overall stage | I | 13 (37.1) | 9 (25.7) | 0.027 |
|  | II | 8 (22.9) | 4 (11.4) |  |
|  | III | 10 (28.6) | 21 (60.0) |  |
|  | IV | 4 (11.4) | 0 (0) |  |
|  | Recurrence | 0 (0) | 1 (2.9 |  |
| Vascular involvement |  | 9 (25.7) | 10 (30.3) | 0.713 |
| CA19-9 at diagnosis (mean± SD, U/ml) |  | 25.4± 19.3 | 1971.8± 4927.0 | 0.022 |
| CEA at diagnosis (mean± SD, ng/ml) |  | 9.2± 24.3 | 31.3± 133.0 | 0.118 |
| ALBI grade | 1 | 17 (50.0) | 8 (22.9) | 0.017 |
|  | 2 | 17 (50.0) | 23 (65.7) |  |
|  | 3 | 0 (0) | 4 (11.4) |  |

| **Supplementary Table 5. Treatment details of low and high CA19-9 blood levels at presentation** | | | |  |
| --- | --- | --- | --- | --- |
|  |  | CA19-9 at diagnosis ≤ 63 U/ml, N=35 (%) | CA19-9 at diagnosis > 63 U/ml, N=35 (%) | P value |
| Dose per fraction | Conventional (180- 200 cGY/Fx) | 12 (34.3) | 16 (45.7) | 0.166 |
|  | Hypofractionated (201- 500 cGY/Fx) | 20 (57.1) | 19 (54.3) |  |
|  | Ultrafractionated (≥501 cGY/Fx) | 3 (8.6) | 0 (0) |  |
| Number of fractions | 1- 5 | 3 (8.6) | 1 (2.9) | 0.493 |
|  | 6- 20 | 10 (28.6) | 13 (37.1) |  |
|  | ≥21 | 22 (62.9) | 21 (60) |  |
| BED_10_ (mean± SD, Gy) |  | 78.9± 12.6 | 71.9± 20.2 | 0.088 |
| Treatment gap | Yes | 3 (8.8) | 7 (20.6) | 0.171 |
| RT treatment modality | Proton | 13 (37.1) | 13 (38.2) | 0.009 |
|  | Photons | 14 (40.0) | 21 (61.8) |  |
|  | Proton and photons | 8 (22.9) | 0 (0) |  |
| Chemotherapy concurrent | Yes | 21 (60.0) | 24 (70.6) | 0.384 |
| Type concurrent chemotherapy | Capecitabine | 16 (69.6) | 17 (81.0) |  |
|  | 5FU | 7 (30.4) | 4 (19.0) |  |
|  | Other | 0 (0) | 0 (0) |  |
| Chemotherapy other than concurrent | None | 16 (45.7) | 15 (42.9) | 0.810 |
|  | Before and/or after RT | 19 (54.3) | 20 (57.1) |  |
| GTV volume (mean± SD, cm³) |  | 167.6± 171.3 | 117.9± 172.7 | 0.284 |
