# Supplementary material for: Clinical outcomes and risk stratification in unresectable biliary tract cancers undergoing radiation therapy
Source: Radiat Oncol. 2024 Aug 1;19:102. doi: 10.1186/s13014-024-02481-y (PMC11293151; doi:10.1186/s13014-024-02481-y)
Supplement: Supplementary file 3 — Supplementary Material 3 [file 13014_2024_2481_MOESM3_ESM.docx]

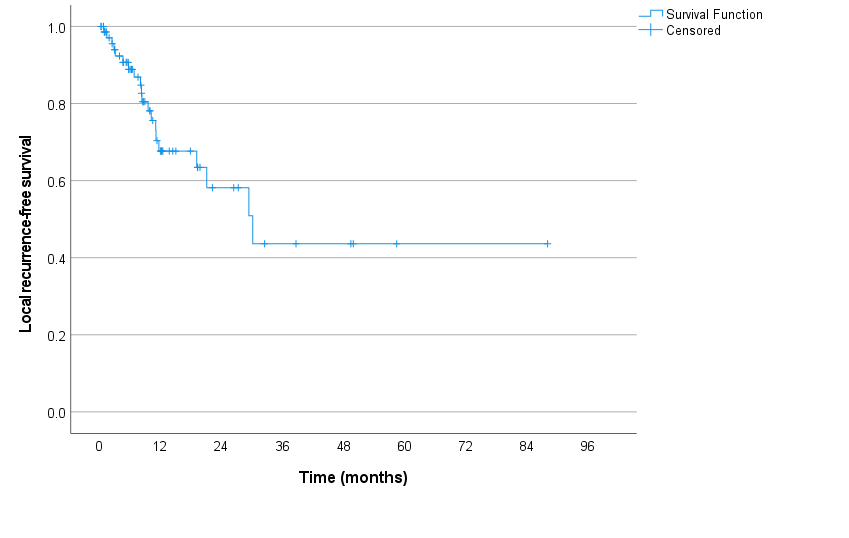


Supplementary Figure 2. Local recurrence-free survival of unresectable BTC patients treated with RT.

| Number at risk | 74 | 25 | 10 | 5 | 4 | 1 | 1 | 1 |
| --- | --- | --- | --- | --- | --- | --- | --- | --- |
